# Supplementary material for: Mechanisms governing the pioneering and redistribution capabilities of the non-classical pioneer PU.1
Source: Nat Commun. 2020 Jan 21;11:402. doi: 10.1038/s41467-019-13960-2 (PMC6972792; doi:10.1038/s41467-019-13960-2)
Supplement: Supplementary file 7 — Source data [file 41467_2019_13960_MOESM7_ESM.zip › Source_Data/FigureS3/FigureS3J_MotifScanOutput/homerResults/motif42.similar.html]

motif42

## Information for motif42

T
A
G
C
A
G
T
C
A
T
C
G
A
T
C
G
A
G
T
C
A
G
T
C
A
G
T
C
  
Reverse Opposite:  

A
C
T
G
C
T
A
G
A
C
T
G
A
T
G
C
T
A
G
C
T
C
A
G
A
T
C
G
  

|  |  |
| --- | --- |
| p-value: | 1e-29 |
| log p-value: | -6.820e+01 |
| Information Content per bp: | 1.809 |
| Number of Target Sequences with motif | 1815.0 |
| Percentage of Target Sequences with motif | 13.75% |
| Number of Background Sequences with motif | 3669.6 |
| Percentage of Background Sequences with motif | 10.05% |
| Average Position of motif in Targets | 614.5 +/- 615.3bp |
| Average Position of motif in Background | 320.9 +/- 176.1bp |
| Strand Bias (log2 ratio + to - strand density) | 0.1 |
| Multiplicity (# of sites on avg that occur together) | 1.68 |
| Motif File: | file (matrix) reverse opposite |

### Similar de novo motifs found

|  |  |  |  |  |  |  |  |
| --- | --- | --- | --- | --- | --- | --- | --- |
| Rank | Match Score | Redundant Motif | P-value | log P-value | % of Targets | % of Background | Motif file |
| 1 | 0.889 | A C T G C T A G A T C G A G T C T G A C C T A G A C T G A T G C G A T C | 1e-19 | -45.514801 | 5.94% | 3.95% | motif file (matrix) |
